# Pancreatitis in pre-adolescent children: a 10 year experience in the pediatric emergency department

**DOI:** 10.1186/s12873-019-0281-y

**Published:** 2019-11-21

**Authors:** Melanie M. Randall, Sarah McDaniels, Kristina Kyle, Meina Michael, Julia Giacopuzzi, Lance A. Brown

**Affiliations:** Department of Emergency Medicine, Loma Linda University Medical Center and Children’s Hospital, 11234 Anderson Street, Loma Linda, CA 92354 USA

**Keywords:** Pancreatitis, Acute pancreatitis, Recurrent pancreatitis, Emergency department, Pre-adolescent

## Abstract

**Background:**

The diagnosis of pediatric pancreatitis has been increasing over the last 15 years but the etiology of this is uncertain. The population of pre-adolescent patients with pancreatitis in the emergency department has not been specifically described. Our objective was to determine the characteristics of these patients to illuminate this population and disease in order to better identify them and avoid a delay in diagnosis and treatment.

**Methods:**

This was a retrospective descriptive study of consecutive pediatric patients under the age of 13 years between 2006 and 2016 who presented to our pediatric emergency department with a diagnosis of atraumatic pancreatitis. Patient characteristics, lab and imaging results, identified etiology of pancreatitis, and recurrence rates were recorded and evaluated.

**Results:**

There were 139 visits, of which 85 were for a first episode of acute pancreatitis, and 54 were patients with an episode of recurrent pancreatitis. The median age for all visits was 8 years (IQ range 5–11). Of the acute cases, 26% had uncertain or undetermined etiologies of which half were thought to likely be viral related; 20% had systemic inflammatory or autoimmune diseases; 19% were associated with medications, with the most common being valproic acid; 16% were cholelithiasis-related; and 15% were found to have a genetic, congenital or structural etiology. No patients had elevated triglycerides. Those with cholelithiasis and genetic or structural defects were found to have a higher recurrence rate than those with other etiologies. There were only four patients diagnosed with chronic pancreatitis.

**Conclusions:**

The etiology of pancreatitis in pre-adolescent children has a different distribution than in adolescents and adults, with gallstone disease less frequent and concurrent contributing illness more common. Patients on pancreatitis-causing medications or with known genetic risk or structural pancreatic problems should be tested for pancreatitis if presenting with concerning symptoms. Hypertriglyceridemia and chronic pancreatitis with evidence of pancreatic exocrine insufficiency is uncommon in this population.

## Background

The diagnosis of pancreatitis in the emergency department has been increasing in the pediatric population over the last 15 years but the etiology of this is uncertain [1–10]. It is possibly due to increase in rates of childhood obesity and subsequent increase in biliary tract disease [9, 11–14]. Studies have shown that biliary disease is usually the most common comorbidity associated with pediatric pancreatitis [2, 15–17]. However, these studies include a wide age range including both young children and adolescents. Another theory about the cause of increased childhood pancreatitis is a heightened awareness of pancreatitis among clinicians and increase in widespread access to lipase testing [1, 6, 7, 9]. There are few studies that focus primarily on younger patients and describe the etiology, risk factors and outcome of these patients, and no studies that look specifically at pre-adolescent children in the emergency department [18].

Traditionally, testing for pancreatitis in children has never been a routine occurrence since pancreatitis was rare in this population. However, with the reported increase in incidence, the larger questions are who needs to be screened for pancreatitis and if the disease has the same associations, course and recurrences as in adults. While adolescents are more comparable to young adults physiologically and likely have many similar risk factors for pancreatitis such as biliary disease, there is very little information on the risk factors, disease presentation, severity and recurrence in the pre-adolescent population.

Our objective was to determine the characteristics of this patient population in order to illuminate this population and disease. With a better description of these children, emergency physicians may be better able to identify them and avoid a delay in diagnosis, which would improve quality of care.

## Methods

We performed a retrospective study of all consecutive pediatric patients under the age of 13 years between 2006 and 2016 who presented to the pediatric emergency department with a final diagnosis of pancreatitis. Our pediatric emergency department at Loma Linda University Medical Center is a tertiary referral center with approximately 30,000 ED visits per year and is the only children’s hospital serving San Bernardino and Riverside counties in Southern California with > 1 million children. An ICD code of acute, recurrent or chronic pancreatitis and age less than 13 years were used to query the medical records database. Children transferred from an outside facility were included. Exclusion criteria included patients 13 years of age or greater, patients with traumatic pancreatitis, and patients with a diagnosis of acute pancreatitis(AP) but without evidence of at least 2 of the following: 1) abdominal pain compatible with AP (abdominal pain of acute onset, especially in the epigastric region), 2) serum amylase and/or lipase ≥3 times upper limits of normal (reference ranges: amylase 20-90 U/L, lipase 10-45 U/L), 3) imaging findings consistent with AP [1]. Charts of patients with a previous history of pancreatitis but currently admitted for an unrelated problem and with no other signs or symptoms of concurrent pancreatitis were also excluded.

Using a standardized data collection form and trained data collectors not blinded to the study objective, the following data points were collected: gender, height, weight, ethnicity, past medical history, medications, and whether or not the patient had a family history of pancreatitis. The following test results were collected: aspartate aminotransferase(AST) level, lipase level, amylase level, total bilirubin level, triglyceride level, ultrasound(US) results, computed tomography(CT) results, endoscopic retrograde cholangiopancreatography(ERCP) results, and magnetic resonance cholangiopancreatography(MRCP) results. Finally, the stated etiology of pancreatitis, whether the episode was acute, chronic, or recurrent, and the number of previous pancreatitis episodes were obtained.

We planned to develop etiologic categories starting with cholelithiasis, triglycerides, and medications. Other categories were determined in a post hoc manner. We assumed that alcohol would not be a significant etiology in this age group due to previous research showing that alcohol use disorder is nonexistent to extremely rare in the preadolescent age range, use of alcohol among children has declined significantly over the last 10 years, and that alcohol has been found to be nearly negligible as an etiology for pediatric pancreatitis [19–25]. Patients with traumatic pancreatitis were identified and excluded as this group has been studied extensively.

Descriptive statistics were used. Detailed descriptions of patients with no known risk factors who were found to have pancreatitis were to be determined. This study was approved by our institutional review board.

## Results

During the 10 year period from 2006 to 2016, we identified 153 visits to the emergency department for children less than 13 years with a final diagnosis of pancreatitis. There were 14 patients diagnosed as having traumatic pancreatitis and these were excluded, leaving 139 patients with nontraumatic pancreatitis.

Of the 139 visits, 85 visits were children with a first episode of acute pancreatitis, and 54 were visits for recurrent pancreatitis. The median age for all visits was 8 years (IQ range 5–11). The median age for acute pancreatitis visits was 8 years (IQ range 4–11). Thirty patients had identified recurrences. The median time from the first acute episode to a first recurrence was 6.3 months (IQ range 2.5–12 months). The median time to any recurrent episode after a previous episode was 6 months (IQ range 3–12 months). Of patients with acute pancreatitis, 35 patients (41%) were male and 50 (59%) were female, and the median lipase level was 881 U/L (IQ range 398–1688). Of the patients with recurrent pancreatitis, 23 patients (43%) were male and 31 (57%) were female, and the median lipase level was 1091 U/L (IQ range 358–2130).

Of the acute pancreatitis visits and their etiologies, we identified seven broad categories in a combination of presumed grouping and post hoc analysis: Cholelithiasis related, Medication induced, Hypertriglyceridemia, Autoimmune or Systemic related, Genetic or Structural, Post-surgical, and Uncertain etiology.

Twenty two (26%) of the acute cases were patients with uncertain/undetermined etiologies of which 13 (15%) were listed as idiopathic and 9 (11%) were possibly infectious (Table 1). One patient in the idiopathic group had one identified recurrence.

**Table 1:** Acute pancreatitis - 85 cases

Etiology	#	Age in yrs Median (IQR/range)	BMI Median (IQR)	Lipase in U/L Median (IQR)	Presumed etiology or Associations	Identified recurrences
**Uncertain - 22 (26%)**						
Idiopathic	13	8 (5.5–10.5 / 1–11)	16.3 (14.6–18.8)	736 (387–888)	–	1 pt. w/hx chronic constipation had 2 recurrences
Possibly Infectious	9	3 (2–10 / 0.9–12)	20.4 (16–20.9)	450 (160–1062)	All thought to be viral related	–
**Systemic - 17 (20%)**						
Associated Acute Systemic	10	6.5 (5–11.25 / 1–12)	16.8 (14.2–22.4)	670 (205–1468)	3 pts. DKA 2 pts. Kawasaki’s disease 1 pt. HUS 1 pt. ATN 1 pt. Takayasu’s arteritis 1 pt. AKI/DI 1 pt. new Leukemia	–
Associated Chronic Systemic	7	7 (3–12 / 2–12)	16.1 (13.4–18.7)	773 (292–1891)	3 pts. Celiac disease 1 pt. Lupus 1 pt. Hypothyroidism 1 pt. Ulcerative colitis 1 pt. Crohn’s	1 pt. w/celiac disease had 2 recurrenced
**Medications - 16 (19%)**						
Valproic Acid	7	9 (8–12 / 6–12)	21.7 (18.7–25.2)	893 (589–1200	–	–
Chemotherapy	4	8.5 (4.25–9.75 / 3–10)	22.6 (16–28)	1717 (800–8296)	3 pts. on pegaspargase 1 pt. on pentamadine	1 pt. on pegaspargase had 3 recurrences
Oxcarbazepine	1	9	18.5	1644	–	–
Metronidazole	1	5	12.4	2064	–	–
Erythromycin	1	12	“Under- weight”	176	–	–
Chlorothiazide	1	1	21	182	–	–
TPN	1	0.5	17.9	265	–	–
**Cholelithiasis - 14 (16%)**						
No past history of gallstones	11	9 (5–11 / 2–12)	26.5 (18.1–33.2)	1954 (1391–3473)	–	3 pts. w/1 recurrence each
Known history of gallstones	3	12 (10–12 / 10–12)	29 (19.7–45)	437 (137–1444)	–	1 pt. w/1 recurrence
**Structural - 7 (8%)**						
Choledochal Cyst	5	4 (2.5–4.5 / 2–5)	16.6 (15–20.3)	1702 (527–9550)	–	–
Caroli disease	1	1	23.6	1683	congenital abnormality of common bile duct	2 recurrences
Pancreatic Duct Stricture	1	4	17	3283	–	4 recurrences
**Genetic - 6 (7%)**						
Propionic Acidemia	3	7 (6–12 / 6–12)	18.3 (14.2–22.4)	2262 (1327–3292)	–	–
Hennekam syndrome	1	11	13.1	197	Pt with Hennekam syndrome and on TPN	1 recurrence
CTRC mutation	1	5	13.7	2000	–	1 recurrence
Heterozygous Cystic Fibrosis	1	9	23.6	290	–	1 recurrence
**Post-operative - 3 (4%)**						
s/p Appendectomy	1	9	19	189	s/p appendectomy, had intra-abd. abscess & lipase was elevated	
s/p Spinal Fusion surgery	1	8	*	178	s/p spinal surgery, abd.pain and emesis, lipase found elevated	2 recurrences
s/p ERCP	1	11	18	651		

*yrs* years

*IQR* interquartile range

*BMI* body mass index

*DKA* diabetic ketoacidosis

*HUS* hemolytic uremic syndrome

*ATN* acute tubular necrosis

*AKI/DI* acute kidney injury / diabetes insipidus

*TPN* total parenteral nutrition

*CTRC* chymotrypsin C

*s/p* status post

*ERCP* endoscopic retrograde cholangiopancreatography

*not available

Seventeen (20%) of acute pancreatitis patients had concurrent or postulated contributing systemic illnesses including acute and chronic inflammatory and autoimmune diseases (Table 1). The only diseases with more than one patient were those with diabetes type one with DKA, Kawasaki’s disease, and Celiac disease. The only identified recurrence was one patient with possible Celiac disease who had two recurrences during the study period.

Sixteen (19%) of acute pancreatitis cases were medication-induced or medication-associated with the most common being valproic acid and chemotherapy. One patient who was on the chemotherapy pegaspargase had an identified recurrence. (Table 1).

Cholelithasis-related etiologies included 14 (16%) of the acute cases with 11 of these having no known history of gallstones. Of the patients with no history of gallstones, nine of the eleven were overweight or obese. Four patients in this group had identified recurrences. (Table 1).

13 (15%) of the patients were found to have a genetic, congenital or structural abnormality included five patients with congenital choledochal cysts, three patients with propionic acidemia, two patients with hereditary pancreatitis, one patient with Hennekam syndrome on TPN, one patient with Caroli disease, and one patient with a possible pancreatic/ampulla duct stricture (Table 1). Five of the 13 patients in this group had identified recurrences during the study period

There were three cases of post-operative pancreatitis after appendectomy, spinal fusion surgery and ERCP, respectively. The patient with spinal fusion had two identified recurrences.

No patients had hypertriglyceridemia during the study period.

There were 54 visits for recurrent pancreatitis of which 20 patients had one episode each of recurrent pancreatitis; six patients each had two episodes of recurrent pancreatitis; one patient had three recurrences; one patient had five recurrences; two patients each had seven recurrences.

Of the 30 patients with recurrent pancreatitis, 16 were found to have underlying genetic etiologies or structural risk factors including 11 patients with genetic variations, one patient treated for autoimmune pancreatitis, one patient with Caroli disease, one patient with Hennakam syndrome, one patient with Celiac disease, and one patient with a pancreatic duct stricture. Four patients of those with recurrent pancreatitis were diagnosed with chronic pancreatitis, three of which were found to have genetic mutations associated with pancreatitis: one with an HT1–12 gene defect and two with a PRSSI mutation.

All patients had pancreatitis determined by an elevated lipase or amylase level and symptoms of pancreatitis except for six patients, who were included in our analysis. Of the six patients, two were diagnosed with chronic pancreatitis and treated for their episodes despite normal lipase levels and no imaging findings of acute pancreatitis; three patients were diagnosed with recurrent acute pancreatitis based on imaging: one with an US showing an echogenic pancreas, one with a CT showing a pseudocyst, and one with an US and CT showing a thinned pancreas and infected pancreatic pseudocyst. The last patient had a known choledochal cyst and was admitted for surgical correction after developing abdominal pain without lipase elevation. Any patients with elevated lipase but without pain or imaging consistent with AP would have been excluded, but there were no patients found in this category.

Regarding location of diagnosis, of the 85 patients with acute pancreatitis, 26 patients (31%) were first seen and diagnosed at an outside community hospital and transferred to the ED, two (2.4%) patients had pancreatitis diagnosed by labs through a primary care physician, two (2.4%) patients were diagnosed by labs at an urgent care, and one (1.2%) patient was diagnosed by labs at an oncology clinic. All other patients were seen first and diagnosed in the pediatric emergency department. Of the 54 patients with recurrent pancreatitis, eight (15%) patients were diagnosed at outside community hospitals, five (9%) patients were diagnosed by labs through a primary care physician, and three (5.5%) patients were diagnosed by labs at an urgent care. The remaining 38 patients were seen and diagnosed first in the pediatric emergency department.

## Discussion

We identified medications, treatments, and certain medical and genetic conditions associated with acute pancreatitis. These included valproic acid, chemotherapy, Celiac disease, choledochal cysts, proprionic acidemia, and cholelithiasis. We were surprised by how many patients were identified as having an unknown or suspected autoimmune/systemic etiology, with these two groups comprising nearly half the cases of acute pancreatitis. However in our study it was extremely rare for any of these patients to have an identified recurrence. Other studies of pediatric pancreatitis showed similar significant proportion of idiopathic cases [26–33]. And while we anticipated that hypertriglyceridemia would be uncommon, it was surprising that none of the patients had elevated triglyceride levels. However, this does correlate with the infrequency of pediatric pancreatitis caused by hypertriglyceridemia in the literature [34]. As suspected, alcohol induced pancreatitis was not determined to be an etiology for pancreatitis in this group. Cholelithiasis remained a significant risk factor for acute and recurrent pancreatitis, but as theorized, represented a much smaller proportion of cases than in adults.

Genetic and structural defects represented a significant percentage of patients with recurrent and chronic pancreatitis. This high frequency is similar to that seen in other studies of pediatric pancreatitis [19, 27, 33, 35]. This highlights the importance of pursuing an investigation for an underlying genetic or structural etiology, especially in those presenting with a recurrent episode of pancreatitis or evidence of chronic pancreatitis.

Limitations to this study were that being retrospective, some data was incomplete or missing, and while most patients did not have any recurrent episodes, it is unknown if they did have a recurrence but were seen at another institution. While the diagnosis of acute pancreatitis is generally straightforward given the widespread use of lipase, it is often more difficult to determine the exact etiology of pancreatitis. Some of the patients with the etiology listed as unknown or uncertain had risk factors such as cholelithiasis or family history that were not listed as the etiology.

Many of the patients presented to surrounding non-pediatric emergency departments and may have had a lipase ordered due to routine abdominal pain lab protocols that are more common in adults. When evaluating the presenting signs, symptoms and chief complaint, there were a group of patients who may have been diagnosed only because of this. Since labs are less routinely required or ordered in children than in adults presenting with abdominal pain, it is possible we may be missing pancreatitis in children without known risk factors. However, since the vast majority of children without risk factors had no known recurrences, the importance of diagnosing these episodes is uncertain.

This paper from one regional pediatric facility supports the suspicion that pre-adolescent pancreatitis has a much different distribution of etiologies than in adults. There were many more patients with systemic disease-related pancreatitis, genetic factors, and medication-related cases. While cholelithiasis was still an important risk factor, it comprised only 16% of acute cases as compared to 29–40% in adults [29, 36]. Another way that pediatric pancreatitis may differ from adult pancreatitis is in recurrence rate. Amongst patients with acute pancreatitis in our study, just 15% of these patients had identified recurrences while the rate of recurrence in adults has been described between 20 and 30% [37, 38].

## Conclusions

The etiology of pancreatitis in pre-adolescent children has a different distribution than in adolescents and adults, with cholelithiasis much less frequent. Certain genetic and structural biliary or pancreatic factors represent a larger proportion of cases in children and a much higher proportion of recurrent pancreatitis. Concurrent autoimmune, systemic disease or viral illness contributing to pancreatitis is also more common than in adults. Patients on pancreatitis-causing medications or with known genetic or structural pancreatic issues should be tested for pancreatitis if presenting with concerning symptoms. Likewise, patients with recurrent pancreatitis should be evaluated for genetic etiologies or structural defects. Patients without pancreatitis risk factors rarely developed recurrent pancreatitis. Hypertriglyceridemia was not found to be an etiology in this study. Chronic pancreatitis with evidence of pancreatic exocrine insufficiency was uncommon.

## Data Availability

The datasets used and analyzed during the current study are available from the corresponding author on reasonable request.

## Abbreviations

AP: Acute pancreatitis
AST: Aspartate aminotransferase
CT: Computed tomography
DKA: Diabetic ketoacidosis
ERCP: Endoscopic retrograde cholangiopancreatography
MRCP: Magnetic resonance cholangiopancreatography
TPN: Total parental nutrition
US: Ultrasound
